# Adsorption Characteristics and Enrichment of Emodin from Marine-Derived *Aspergillus flavipes* HN4-13 Extract by Macroporous Resin XAD-16

**DOI:** 10.3390/md20040231

**Published:** 2022-03-28

**Authors:** Lizhi Gong, Yuzhen Wu, Xiaohan Qiu, Xiujuan Xin, Faliang An, Miaomiao Guo

**Affiliations:** 1 Key Laboratory of Cosmetic, China National Light Industry, Beijing Technology and Business University, No. 11/33, Fucheng Road, Beijing 100048, China; y30191249@mail.ecust.edu.cn; 2 State Key Laboratory of Bioreactor Engineering, East China University of Science and Technology, 130 Meilong Road, Shanghai 200237, China; y30200462@mail.ecust.edu.cn (Y.W.); y30180978@mail.ecust.edu.cn (X.Q.); xinxj@ecust.edu.cn (X.X.)

**Keywords:** *Aspergillus flavipes* HN4-13, emodin, macroporous resin XAD-16, adsorption kinetics and isotherms, enrichment

## Abstract

Emodin, a hydroxyanthraquinone derivative, has been used as medicine for more than 2000 years due to its extensive pharmacological activities. Large-scale production of emodin has been achieved by optimizing the fermentation conditions of marine-derived *Aspergillus flavus* HN4-13 in a previous study. However, the fermentation broth contained complex unknown components, which adversely affected the study of emodin. Herein, the conditions for the enrichment of emodin from *A*. *flavipes* HN4-13 extract using XAD-16 resin were optimized, and a separation method with high efficiency, simple operation, a low cost, and a large preparative scale was established. The adsorption process of emodin on the XAD-16 resin conformed to pseudo-second-order kinetics and Langmuir models. The optimal conditions for the adsorption process were as follows: An emodin concentration, flow rate, and loading volume of 0.112 mg/mL, 2 BV/h, and 10 BV, respectively. For desorption, 50% ethanol was used to elute impurities and 80% ethanol was used to desorb emodin. After enrichment with XAD-16 resin, the emodin content increased from 1.16% to 11.48%, and the recovery rate was 75.53% after one-step treatment. These results demonstrate the efficiency of the simple adsorption–desorption strategy, using the XAD-16 resin for emodin enrichment.

## 1. Introduction

**Figure 1:**
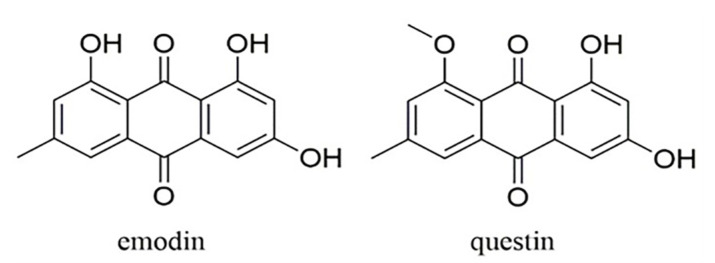
Chemical structures of emodin and questin.

Emodin (1,3,8-trihydroxy-6-methylanthraquinone) is a hydroxyanthraquinone derivative with a variety of activities, including cathartic, anticancer [1], anti-inflammatory, antioxidant [2], antibacterial [3], and anti-diabetic properties [4]. Previous research has indicated that emodin can be used as a potential drug for the prevention and treatment of diseases, such as asthma [5], osteoarthritis [6], Alzheimer’s disease (AD) [7], hepatic disease [8], and cancer [9,10,11]. Emodin is also used as an industrial additive in food and cosmetics [12]. As mentioned above, emodin has broad application prospects and, thus, a high market demand. Questin, a derivative of emodin, with antibacterial activity, can be converted into emodin via a demethylation reaction [13]. The chemical structures of emodin and questin are shown in Figure 1.

At present, emodin is mainly separated from the traditional Chinese medicinal herb *Rheum palmatum Linn* [12]. Disadvantageously, however, the plant requires a long period, and a complex process is required to separate emodin from the plant extract, with a low recovery rate [14]. The chemical synthesis of emodin has the disadvantages of low yields and a complex route. Chalothorn et al. synthesized emodin in an eight-step complex chemical reaction with a total yield of only 37% [15]. Recently, microorganisms have been found to be a rich source of bioactive metabolites, and emodin is also reportedly contained in the secondary metabolites of fungi from *Aspergillus* and *Penicillium* genera [13,16]. In a previous experiment in our lab, by optimizing the fermentation conditions of marine-derived *A*. *flavipes* HN4-13 in KH_2_PO_4_-supplemented medium, the yield of emodin was increased ten-fold (to 185.56 mg/L) at the shake flask level [17], which is conducive to large-scale production of emodin. However, the fermentation broth contained complex unknown components that adversely affected the study of the biological activity of emodin. Therefore, it is highly desirable to establish a simple and efficient method to enrich emodin from *A*. *flavipes* HN4-13 extract.

Macroporous resins have the advantages of a large specific surface area, strong adsorption, stable physical and chemical properties, a fast adsorption rate, excellent selectivity, high repeatability, and low cost [18]. These resins are widely used in the production of medicines and natural products [19,20], making up for the deficiencies of traditional membrane-separation technology and solvent-extraction methods. Amberlite XAD resins are styrene–divinylbenzene copolymers, which mainly adsorb organic matters through hydrophobic bonds [21]. Among them, XAD-16 resin has a good adsorption capacity for weakly polar compounds with its pore size of 200 Å and surface area of 800 m^2^/g [22]. Compared with some modified adsorbents, XAD-16 resin is more suitable for industrial large-scale production due to its lower cost and easier access [23,24]. Therefore, in this study, a systematic separation process using macroporous resin was designed and developed to obtain sufficient emodin. The separation process was optimized including the static and dynamic adsorption and desorption variables. Furthermore, to improve the emodin content, questin in the desorption solution was transferred into emodin by a one-step demethylation reaction [15]. Overall, a simply executed, low-cost method with high separation efficiency was developed for emodin enrichment. Herein, we report the systematic separation process for the large-scale production of emodin from fermented *A*. *flavipes* HN4-13 extract. This study laid the theoretical foundation for the industrial application of emodin and was of great significance to the further research and application of emodin activity.

## 2. Results

### 2.1. Resin Screening

**Figure 2:**
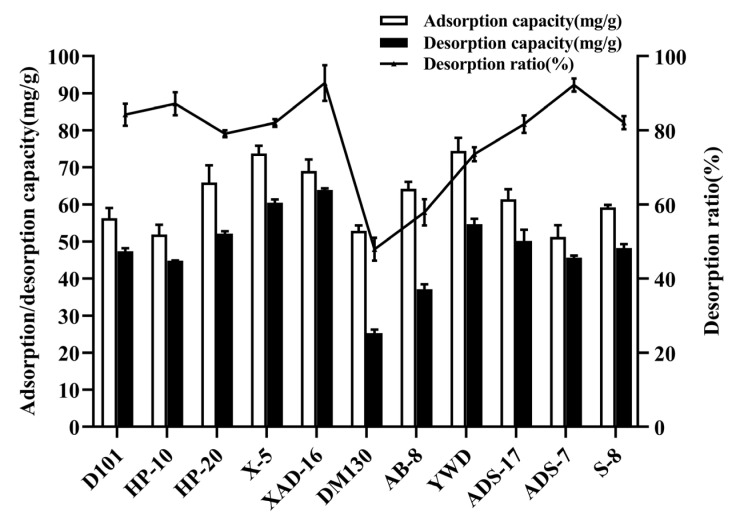
Resin screening for enrichment of emodin. Initial concentration of emodin in the solution: 0.390 mg/mL; desorption solution: 95% ethanol (*v/v*); operating temperature: 25 °C; shaking speed: 120 rpm. Results are the mean of three determinations.

The characteristics of 11 resins with different polarities and pore sizes for emodin adsorption are compared in Figure 2. Among them, XAD-16 resin had a relatively high adsorption capacity and maximum desorption capacity, with values of 73.73 mg/g and 63.91 mg/g, respectively. From the resin parameters in Table 1 and the experimental data in Figure 2, it was deduced that the polarity of the resin greatly affected the adsorption and desorption processes, consistent with a previous report [25]. Emodin is a weakly polar compound and considering the principle underlying the adsorption for compounds on resins, the adsorption capacity of weakly polar and non-polar resins for emodin is higher than that of polar resins [26]. Therefore, the emodin adsorption capacities of HP-20, X-5, XAD-16, YWD, and AB-8 were generally superior to those of S-8 and ADS-7 as polar resins. In addition, resins with larger pore sizes and surface area tend to have higher mass exchange rates, and thus, higher desorption and recovery rates [25]. XAD-16 has a large specific surface area of 800 m^2^/g and a pore size of 15 nm, which can also improve the adsorption capacity of emodin. Therefore, XAD-16 was selected for the enrichment of emodin. The application of XAD-16 in natural product separation and enrichment has been widely reported [27,28].

### 2.2. Effect of the Solution pH on XAD-16 Resin Adsorption Capacity

**Figure 3:**
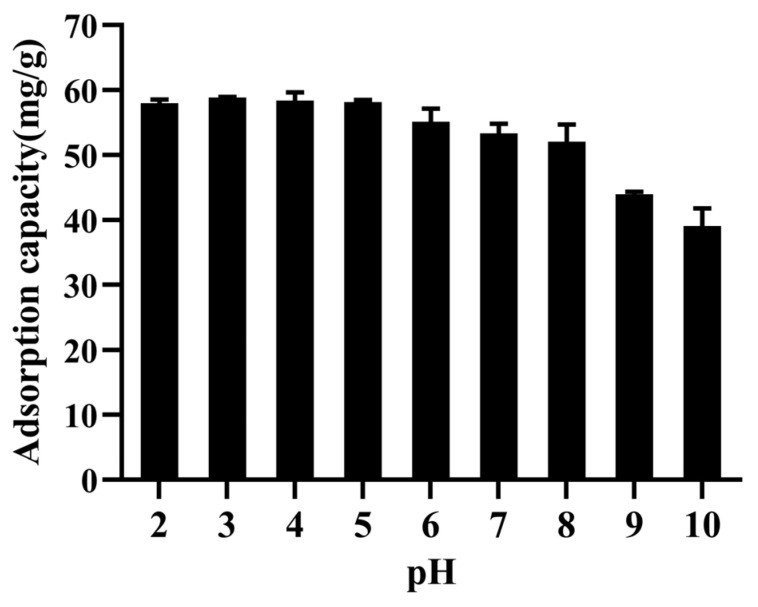
Effect of the solution pH on XAD-16 resin adsorption capacity. Initial concentration of emodin in the solution: 0.3900 mg/mL; operating temperature: 25 °C; shaking speed: 120 rpm. Results are the mean of three determinations.

As shown in Figure 3, the adsorption capacity of the XAD-16 resin for emodin was the highest at pH 3.0, and the adsorption capacity under acidic conditions was higher than that under alkaline conditions. Emodin was mainly combined with XAD-16 resin through hydrophobic interactions. In the alkaline condition, as a phenolic acid compound, the phenolic hydroxyl of emodin was dissociated into the corresponding anionic form, resulting in the weakened hydrophobic interaction with the resin [29]. However, dissociation was inhibited under acidic conditions. Therefore, the optimum pH for the adsorption process was 3.0, indicating that there was no need to adjust the pH of the sample solution.

### 2.3. Adsorption Kinetics Experiments

**Figure 4:**
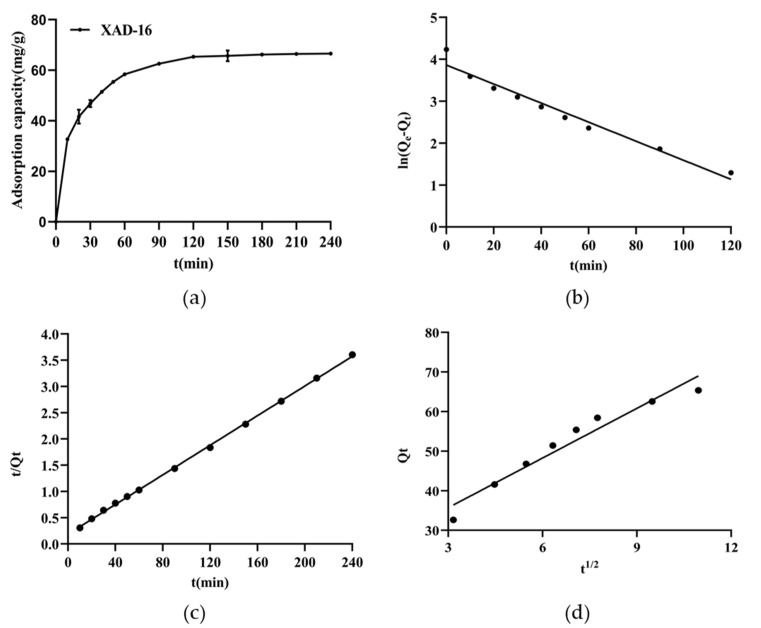
(**a**) The static adsorption kinetic curve of emodin on XAD-16 resin; (**b**) the linear plots of pseudo-first-order kinetics; (**c**) the linear plots of pseudo-second-order kinetics; (**d**) the linear plots of Weber and Morris intra-particle diffusion models. Initial concentration of emodin in the solution: 0.390 mg/mL; desorption solution: 95% ethanol (*v/v*); operating temperature: 25 °C; shaking speed: 120 rpm. Results in (**a**) are the mean of three determinations.

The adsorption kinetics curve can be used to characterize the adsorption efficiency and capacity of the resin when the adsorption process reaches equilibrium. The data reflect the adsorption of the solute on the resin during different time periods. The kinetics curve for the adsorption of emodin on the XAD-16 resin was shown in Figure 4a. In the first 60 min of the adsorption process, the adsorption capacity increased rapidly, and then the growth rate continued to slow down. After 120 min, the adsorption of emodin on the resin reached equilibrium. Consequently, 120 min was selected as the optimal static adsorption time.

To clarify the process of emodin adsorption on XAD-16, the experimental data were fitted with pseudo-first-order kinetics, pseudo-second-order kinetics, and Weber and Morris intra-particle diffusion kinetics models. The results were shown in Figure 4b–d. The relevant parameters for the three models summarized in Table 2 indicated that the *Q_e_* value of the pseudo-second-order kinetic model is much more in line with the experimental *Q_e_* value (63.91 mg/g), indicating that the rate-determining step in the adsorption process is related to the concentrations of both emodin and resin [30]. Moreover, the *R*^2^ value of the pseudo-second-order kinetic model is higher than the *R*^2^ value of the pseudo-first-order kinetics model, which also demonstrates that the pseudo-second-order kinetics model is more appropriate for describing emodin adsorption on XAD-16. For the Weber and Morris intra-particle diffusion kinetics model, the straight line did not cross the origin of coordinates, indicating that the adsorption process of emodin on XAD-16 was affected by many factors, such as interparticle diffusion and boundary layer diffusion [31].

### 2.4. Adsorption Isotherms and Thermodynamic Experiments

**Figure 5:**
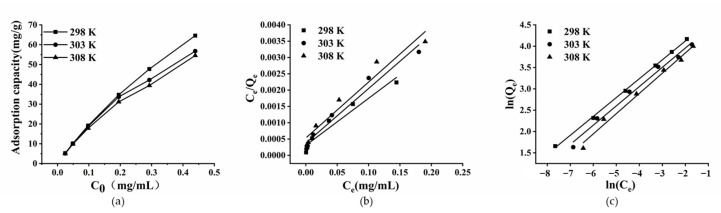
(**a**) Adsorption isotherms of emodin on XAD-16 at 298, 303 and 308 K; (**b**) the linear plots of Langmuir model; (**c**) the linear plots of Freundlich model. Initial concentrations of emodin in adsorption solution: 0.024, 0.049, 0.112, 0.195, 0.293, and 0.439 mg/mL; shaking speed: 120 rpm.

To interpret the process of emodin adsorption on the XAD-16 resin more comprehensively, the adsorption isotherms and thermodynamic models were established. As shown in Figure 5a, with an increase in the initial concentration of emodin in the solution, the adsorption capacity of XAD-16 increased, but the increasing trend was continuously alleviated. It was speculated that the adsorption of emodin on XAD-16 tended to be saturated, so increasing the concentration of emodin could not improve the adsorption capacity of the resin. Therefore, the initial concentration of emodin used in subsequent experiments was 0.112 mg/mL. When the initial concentration of emodin was fixed, the adsorption capacity of resin increased with decreasing temperature. At 298 K, the adsorption capacity of XAD-16 for emodin was higher than that at 303 K and 308 K, indicating that the adsorption process was exothermic. In consideration of the experimental results and the actual operating conditions, 298 K was selected as the optimal operating temperature.

Adsorption isotherms are used to describe the distribution of solutes in solvents and adsorbents. They are related to the characteristics of the adsorbents and the interactions between the adsorbents and the solutes [32]. The Langmuir model assumes that the target absorbed on the adsorbent is a single layer and that there is no interaction with the solute. However, the Freundlich isotherm model describes multi-layer adsorption, assuming that the ratio of the solute adsorbed on the adsorbent to the concentration of the solute in the solution exists as a function of the solution concentration [33]. As shown in Figure 5b,c, the Langmuir and Freundlich models were used to explain the thermodynamics of emodin adsorption on XAD-16. The relevant parameters of the two models at 298, 303, and 308 K were summarized in Table 3. The *R*^2^ values of the Freundlich model were higher than that of the Langmuir model at the same temperatures, which implied that the adsorption process of emodin on XAD-16 was more in line with the Freundlich model and it belonged to multi-layer adsorption. The *K_F_* value decreased with the increase in temperature, suggesting that the adsorption process was exothermic. The 1/*n* value was within 0.1–0.5, indicating that the adsorption of emodin on XAD-16 was relatively facile.

The relevant adsorption thermodynamic parameters at 298, 303, and 308 K are shown in Table 3. ∆*G* < 0 implied that the adsorption process was spontaneous and feasible. In addition, the absolute value of ∆*G* decreased with increasing temperature, indicating that low temperature was more conducive to the adsorption process [34,35]. ∆*H* < 0 indicated that the adsorption process was exothermic, and ∆*H* < 43 kJ/mol showed that the process was a physical adsorption process [36,37]. ∆*S* < 0 suggested that the process is a process with a reduced degree of system confusion.

### 2.5. Dynamic Breakthrough Curves of Emodin on XAD-16

**Figure 6:**
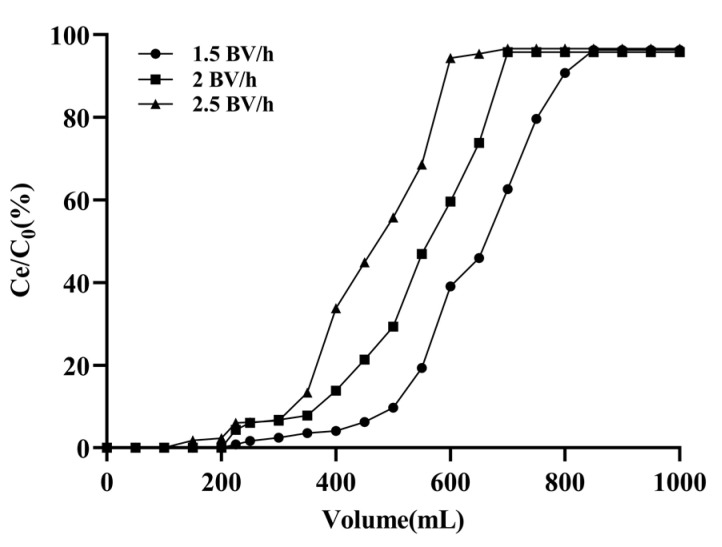
Dynamic breakthrough curve of emodin on XAD-16. Initial concentration of emodin in adsorption solution was 0.112 mg/mL.

By studying the dynamic adsorption process, the dynamic breakthrough curve was constructed, and the sample volume and loading flow rate were optimized. As shown in Figure 6, a faster loading flow rate (2.5 BV/h) was conducive to reducing the time required for the resin to reach the breakthrough point (the emodin concentration in the effluent was 5% of the initial concentration), but the adsorption capacity of the XAD-16 for emodin was decreased. Therefore, a low flow rate was superior to a high flow rate, but the too-low flow rate (1.5 BV/h) would prolong the experimental operation time and greatly increase the loading volume, which was inconsistent with the concept of green production for environmental protection. Hence, 2 BV/h was selected as the optimal loading flow rate, and the loading volume was 200 mL (10 BV).

### 2.6. Optimal Eluent Concentration and Flow Rate for Dynamic Desorption

**Figure 7:**
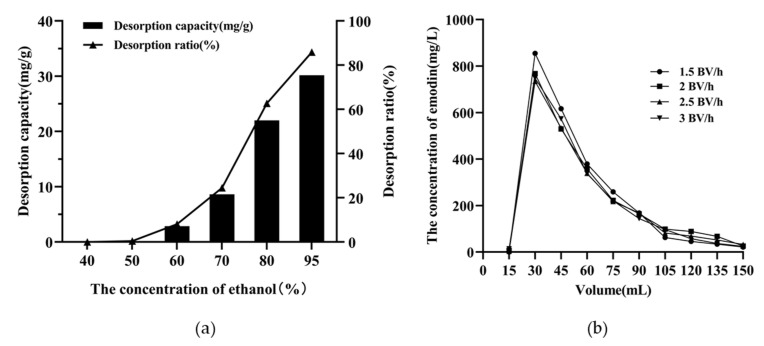
(**a**) The selection of eluent concentration of emodin on XAD-16; (**b**) dynamic desorption curve of emodin on XAD-16.

The elution efficiency of emodin adsorbed on resin varied with the concentration of ethanol solution. As shown in Figure 7a, the emodin content in the desorption solution was low when 40% or 50% ethanol was used as the eluent. With an increase in the ethanol concentration from 70% to 80%, the desorption amount and desorption rate of emodin increased significantly, and the impurity content in the eluate was relatively low. Therefore, in this experiment, a 50% ethanol solution was used as the eluent to elute impurities, and an 80% ethanol solution was used as the desorption solution.

When the adsorption of emodin on XAD-16 reached equilibrium, the resin column was first rinsed with 6 BV deionized water, followed by 6 BV 50% ethanol solution to remove the impurities. Subsequently, elution was performed using 80% ethanol at flow rates of 1.5, 2.0, 2.5, and 3.0 BV/h, respectively. As shown in Figure 7b, the emodin content in the eluent was the highest at a flow rate of 1.5 BV/h, and the emodin was easily collected in the range of 2–6 BV.

### 2.7. Optimal Reaction Time for Conversion of Questin to Emodin

**Figure 8:**
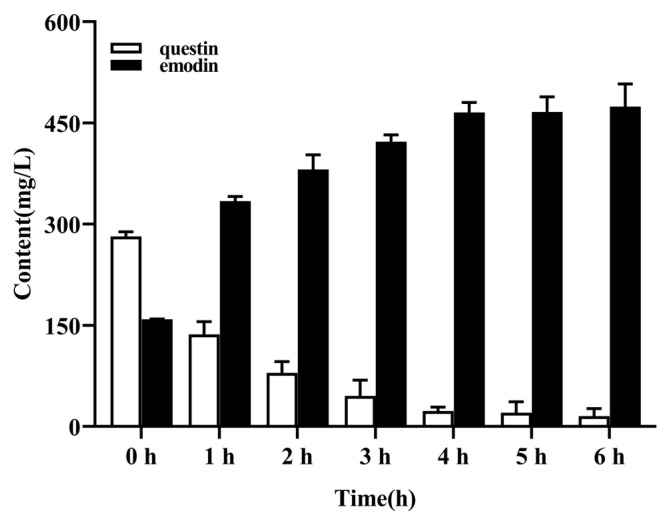
The optimal reaction time for the conversion of questin to emodin. Results are the mean of three determinations.

Questin is a derivative of emodin, and both have similar physicochemical properties. The two compounds could not be separated through adsorption on the XAD-16 resin and subsequent desorption, which reduced the purity of emodin in the desorption solution. Previous experiments have shown that questin can be easily and efficiently converted to emodin via a one-step chemical reaction [17]. As shown in Figure 8, when the reaction lasted for 5 h, questin in the eluate had been essentially converted to emodin.

### 2.8. Purification of Emodin

**Figure 9:**
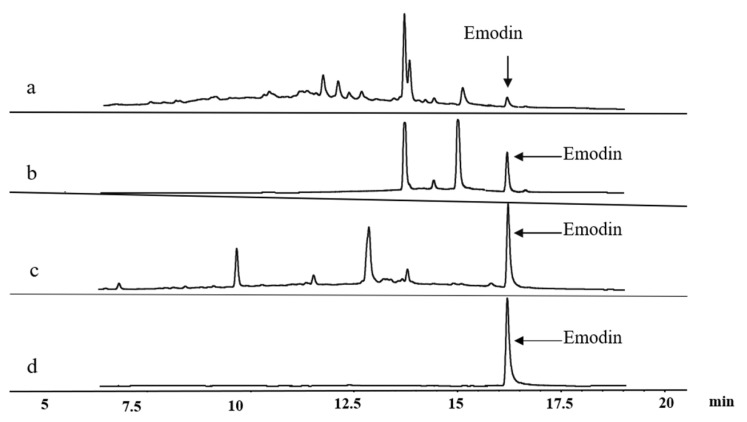
HPLC chromatograms of samples (**a**: Initial; **b**: Resin enrichment; **c**: Reaction conversion; **d**: Further purification).

As shown in Figure 9, after enrichment using XAD-16 resin, the percentage of emodin increased from 1.16% to 11.48% with a recovery of 75.53%, which was an approximately ten-fold increase. The questin content increased from 3.70% to 18.43% with the recovery of 58.45%, representing an approximately five-fold increase. After chemical transformation, the emodin content increased to 25.75%, with a recovery of 50.32%. A total of 32.5 mg of emodin crystals was obtained with a purity of 94.4% through successive resin enrichment, chemical conversion, separation, and purification on a silica gel column and reversed-phase column. The ^1^H NMR and HRESIMS data of the purified emodin were detailed in the Supporting Information (Figures S1 and S2).

## 3. Discussion

In summary, XAD-16 resin was selected for the enrichment of emodin from the fermentation broth of *A*. *flavipes* HN4-13. The static and dynamic parameters were optimized using the kinetic and isothermal models. High-purity emodin (94.4%), with a recovery of 75.53%, was obtained under the optimized conditions. The percentage of emodin in the *A*. *flavipes* HN4-13 extract reached 29.91% after enrichment with XAD-16 resin and chemical transformation, representing a more than 25-fold increase. Meanwhile, the majority of complex unknown components were removed, which was conducive to further study on the activity of emodin. Hence, the XAD-16 resin was shown as an effective and promising tool for the large-scale separation of emodin due to its high adsorption and recovery characteristics. In addition, the regeneration and stability of the adsorbent are two of the important parameters in evaluating solid-phase efficiency for adsorption studies [38]. In previous studies, Liu et al. have evaluated the reusability of XAD-16 resin and demonstrated its advantages of easy regeneration [27]. Therefore, a separation method with high separation efficiency, simple operation, and low cost for the enrichment of emodin from *A*. *flavipes* HN4-13 fermentation broth was successfully established, which may provide a theoretical basis for the industrial-scale production of emodin.

## 4. Materials and Methods

### 4.1. Materials and Reagents

The chemical reagents of analytical grade were purchased from Shanghai Titan Technology Co., Ltd. (Shanghai, China). Standard emodin and questin were isolated from the laboratory.

The macroporous resins D101, HP-10, HP-20, X-5, XAD-16, DM130, AB-8, YWD, ADS-17, ADS-7m, and S-8 were purchased from Nankai Hecheng Technology Co., Ltd. (Tianjin, China). The resins were pretreated with 95% ethanol for 24 h, then sequentially treated with 5% HCl, 5% NaOH, and deionized water, and finally dried at 50 °C to a constant weight. Prior to the experiments, the resins were kept in 95% ethanol for 24 h to activate and then were rinsed with deionized water thoroughly. The main physical properties of these resins are summarized in Table 1.

### 4.2. Preparation of Sample Solution

The fermentation medium used for *A*. *flavipes* HN4-13 was soluble starch, yeast extract, sea salt, KH_2_PO_4_, MgSO_4_·7H_2_O, and FeSO_4_·7H_2_O, which was consistent with the emodin fermentation process reported previously [17]. The fermentation broth was concentrated by filtration, and the concentrate was extracted three times with an equal volume of ethyl acetate (EtOAc). The EtOAc extract was subjected to rotary evaporation under vacuum conditions (−0.08 MPa, 45 °C) to remove EtOAc and obtain the crude extract of the mycelia. We dissolved the crude extract completely with a suitable amount of methanol and filtered out the impurities. Methanol was then removed by a vacuum rotary evaporator to obtain the final extract. The content of emodin in the extracted sample was 1.16%, which was determined by HPLC. The sample solution used in the resin adsorption experiment was prepared by adding 40% aqueous ethanol to the extract.

### 4.3. Static Adsorption and Desorption Experiments

#### 4.3.1. Resin Screening

Different pretreated resins (each 1 g) were separately added to 250 mL flasks containing 50 mL of the sample solution (emodin concentration of 0.390 mg/mL). The flasks were shaken (120 rpm) in a constant temperature incubator for 12 h at a room temperature of 25 °C. Once the adsorption equilibrium was reached, the resins were collected by filtration and rinsed with deionized water to remove the residual sample solution. Then, for desorption, the resins were transferred to 50 mL of 95% (*v/v*) ethanol and shaken (120 rpm) for 12 h at 25 °C. The content of emodin in the solutions before and after desorption was analyzed by HPLC. The adsorption capacity (*Q_e_*, Equation (1), desorption capacity (*Q_d_*, Equation (2)) and the desorption rate (*D*, Equation (3)) were calculated by the following equations:

(1) $$Q_{e}=\frac{\left(C_{0}-C_{e}\right)V_{i}}{W}$$

(2) $$Q_{d}=\frac{C_{d}V_{d}}{W}$$

(3) $$D=\frac{C_{d}V_{d}}{\left(C_{0}-C_{e}\right)V_{i}}\times 100\%$$

where *Q_e_* (mg/g) and *Q_d_* (mg/g) are the equilibrium adsorption capacity and desorption capacity, respectively; *C*_0_ and *C_e_* (mg/mL) are the initial concentration and equilibrium concentration of emodin in the solution, respectively; *V_i_* and *V_d_* (mL) represent the initial sample solution volume and desorption solution volume, respectively; *W* (g) is the dry weight of the resin; *C_d_* represents the concentration of emodin in the desorption solution; *D* (%) is the desorption ratio.

#### 4.3.2. Effect of pH on Adsorption Capacity

Pretreated resins (each 1 g; wet weight) were added to 250 mL flasks containing 50 mL of the sample solution (emodin concentration 0.390 mg/mL), and the pH of the solution was adjusted with hydrochloric acid and sodium hydroxide (initial pH values were 2, 3, 4, 5, 6, 7, 8, 9, and 10, respectively). Then, the flasks were shaken (120 rpm) for 12 h at 25 °C, and the content of emodin in the solutions was analyzed by HPLC when the adsorption process reached equilibrium.

#### 4.3.3. Adsorption Kinetics Experiments

Pretreated resins (each 1 g; wet weight) were added to 250 mL flasks containing 50 mL of the sample solution (emodin concentration 0.390 mg/mL). Then, the flasks were shaken (120 rpm) for 12 h at 25 °C, and the content of emodin in the solutions during the adsorption process was analyzed by HPLC at regular intervals (30, 60, 90, 120, 150, 180, 210, and 240 min). The pseudo-first-order model (4) and pseudo-second-order model (5) were used to describe the adsorption process. The linear forms of them can be given as:

(4) $$\log(Q_{e}-Q_{t})=\log Q_{e}-\frac{k_{1}}{2.303}t,$$

(5) $$\frac{t}{Q_{t}}=\frac{1}{k_{2}Q_{e}{}^{2}}+\frac{t}{Q_{e}},$$

where *Q_e_* (mg/g) is the equilibrium adsorption capacity, *Q_t_* (mg/g) is the adsorption capacity at different times; *k*_1_ (min^−1^) and *k*_2_ [g/(mg·min)] are the adsorption rate constants of the two models, respectively.

The Weber Morris intra-particle diffusion model (6) was used to analyze the control steps in the adsorption process, which can be given as:

(6) $$Q_{t}=k_{t}t^{1/2}+C,$$

where *Q_t_* has the same meaning as above; *k_t_* [mg/(g·min^1/2^)] is the diffusion rate constant; *C* (mg/g) is the constant related to the boundary layer and thickness; and *t* (min) represents the time.

#### 4.3.4. Adsorption Isotherms and Thermodynamics Experiments

One gram of pretreated resin (wet weight) was added to 250 mL flasks containing 50 mL of the sample solution (the *C*_0_ of emodin were 0.024, 0.048, 0.112, 0.195, 0.293, and 0.439 mg/mL, respectively). The flasks were shaken continuously (120 rpm) for 12 h at 298 K, 303 K, and 308 K, respectively. The emodin concentration in the solutions was analyzed by HPLC when the adsorption process reached equilibrium. The Langmuir model (7) and Freundlich model (8) were used to investigate the adsorption characteristics:

(7) $$Q_{e}=\frac{Q_{m}\;K_{L}C_{e}}{1-K_{L}C_{e}{}^{1/n}}$$

(8) $$Q_{e}=K_{F}C_{e}{}^{1/n}$$

where *Q_e_* (mg/g) and *C_e_* (mg/mL) have the same meanings as above; *Q_m_* (mg/g) is the maximum adsorption capacity; *K_L_* (L/mg) represents the Langmuir constant; *K_F_* ([(mg/g) (L/mg)^1/n^]) represents the Freundlich constant, which will decrease with the increase of temperature; and 1/*n* is an empirical constant related to the adsorption strength.

### 4.4. Dynamic Adsorption and Desorption Experiments

These glass columns (1.5 cm × 30 cm) filled with 12 g of pretreated wet resin (1 BV = 20 mL) were utilized to perform dynamic adsorption and desorption experiments. The resin columns were washed with deionized water to remove the bubbles. The sample solution (emodin concentration 0.112 mg/mL) passed through the resin columns at different flow rates (1.5, 2.0, and 2.5 BV/h). The concentration values of emodin in the desorption solutions were continuously recorded, and the optimal adsorption flow rate was selected based on the maximum adsorption capacity of emodin on the resin column. After adsorption reached equilibrium, the resin columns were rinsed with 6 BV deionized water followed by desorption with different concentrations of ethanol (40%, 50%, 60%, 70%, 80%, and 95%) at a flow rate of 2 BV/h. The eluents were collected and analyzed by HPLC to investigate the effect of ethanol concentration on the desorption process. To select the optimal elution flow rate, the resin columns were successively rinsed with 6 BV deionized water and 6 BV 50% ethanol after the adsorption reached equilibrium. Emodin was then eluted with 10 BV 80% ethanol at flow rates of 1.5, 2.0, 2.5, and 3.0 BV/h, and the desorption solutions were analyzed by HPLC.

### 4.5. Demethylation of Questin

In order to obtain emodin with a higher content, questin in the desorption solution was demethylated and converted into emodin under acidic conditions [15]. The reaction was carried out as follows: 100 mg of the dried eluate was dissolved in 60 mL of glacial acetic acid, and 25 mL of HBr was added. The reaction was heated in a water bath at 85 °C. The concentrations of emodin during the reaction process were analyzed at regular intervals (1, 2, 3, 4, 5, and 6 h).

### 4.6. HPLC Analysis of Emodin and Questin

The HPLC condition for emodin analysis was as follows: The stationary phase was Agilent ZORBAX Eclipse XDB-C18 column (4.6 × 250 mm, 5 μm); the detector was a DAD G1315B UV detector; the detection wavelength was 310 nm; the column temperature was 30 °C; the flow rate was 0.8 mL/min; the mobile phases were methanol with 0.1% formic acid (*v/v*); and the injection volume was 5 μL.

## Figures and Tables

**Table 1:** Physical and chemical properties of various resins.

Resin	Polarity	Surface Area (m^2^/g)	Average Pore Diameter (nm)	Particle Diameter (mm)
D101	non-polar	400–600	10–12	0.2–0.6
HP-10	non-polar	500–650	15–50	0.6–0.7
HP-20	non-polar	850–1000	25–50	0.6–0.7
X-5	non-polar	500–600	29–30	0.3–1.25
XAD-16	non-polar	800	15	0.7
DM130	weak polar	100	25–30	0.3–1.25
AB-8	weak polar	480–520	13–14	0.3–1.25
YWD	weak polar	500–550	9–10	0.3–1.2
ADS-17	middle polar	90–150	25–30	0.3–1.25
ADS-7	polar	100	25–30	0.3–1.25
S-8	polar	100–120	28–30	0.3–1.25

**Table 2:** Kinetic parameters for adsorption of emodin on XAD-16 resin.

Kinetic Model	Equation	*R* ^2^	Parameters
Pseudo-first-order model	$\ln Q_{e}-Q_{t}=3.864-0.0227t$	0.9652	$k_{1}=0.052$ ${Q_{e}}^{\mathrm{cal}}=47.66\;\mathrm{mg}/g$
Pseudo-second-order model	$\frac{t}{Q_{t}}=0.01411t+0.1870$	0.9995	$k_{2}=0.00107$ ${Q_{e}}^{\mathrm{cal}}=70.87\;\mathrm{mg}/g$
Weber and Morris intra-particle diffusion model	$Q_{t}=4.184t^{\frac{1}{2}}+23.17$	0.9459	$k_{t}=4.184$ C=23.17

**Table 3:** Langmuir and Freundlich parameters of emodin on XAD-16.

T (K)	Langmuir Model			Freundlich Model			∆G	∆H	∆S
	*Q_m_*	*K_L_*	*R* ^2^	*K_F_*	1*/n*	*R* ^2^	(kJ/mol)	(kJ/mol)	(kJ/mol)
298	69.44	48.00	0.9215	149.5	0.4421	0.9993	−9.591	−22.97	−0.045
303	59.88	41.75	0.9702	129.3	0.4522	0.9896	−9.401		
308	58.14	34.40	0.9624	120.3	0.4742	0.9895	−9.060		

## Data Availability

Not applicable.

## Supplementary Materials

The following supporting information can be downloaded at: https://www.mdpi.com/article/10.3390/md20040231/s1, Figures S1 and S2: ^1^H NMR and HRESIMS spectra of the purified emodin.
