# Supplementary material for: Adsorption Characteristics and Enrichment of Emodin from Marine-Derived Aspergillus flavipes HN4-13 Extract by Macroporous Resin XAD-16
Source: Mar Drugs. 2022 Mar 28;20(4):231. doi: 10.3390/md20040231 (PMC9028206; doi:10.3390/md20040231)
Supplement: Supplementary file 1 [file marinedrugs-20-00231-s001.zip › marinedrugs-1638554-supplementary.pdf]

Supporting Information

Adsorption Characteristics and Enrichment of Emodin from Marine-Derived *Aspergillus flavipes* HN4-13 Extract by Macroporous Resin XAD-16

Lizhi Gong [1,2], Yuzhen Wu [2], Xiaohan Qiu [2], Xiujuan Xin [2], Faliang An [2,*] and Miaomiao Guo [1,*]

[1] Key Laboratory of Cosmetic, China National Light Industry, Beijing Technology and Business University, No. 11/33, Fucheng Road, Beijing 100048, China

[2] State Key Laboratory of Bioreactor Engineering, East China University of Science and Technology, 130 Meilong Road, Shanghai 200237, China; y30191249@mail.ecust.edu.cn (L.G.); y30200462@mail.ecust.edu.cn (Y.W.); y30180978@mail.ecust.edu.cn (X.Q.); xinxj@ecust.edu.cn (X.X.)

* Correspondence: flan2016@ecust.edu.cn (F.A.); guomiaomiao7@163.com (M.G.);
Tel.: +86-21-6425-3823 (F.A.); +86-010-68987110 (M.G.)

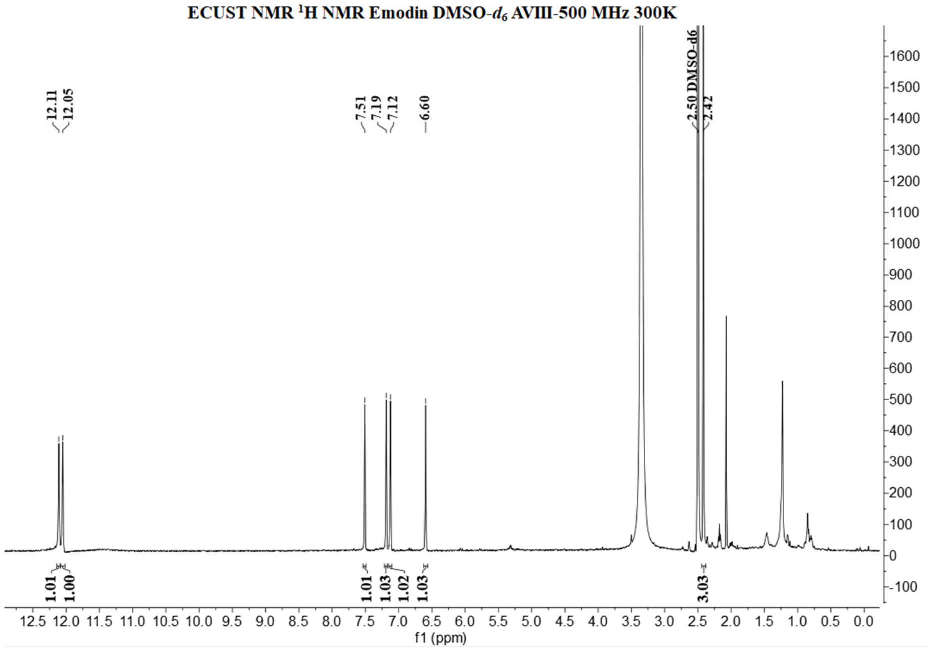

Figure S1 ^{1}H NMR spectrum (500 MHz) of emodin in DMSO-d_6

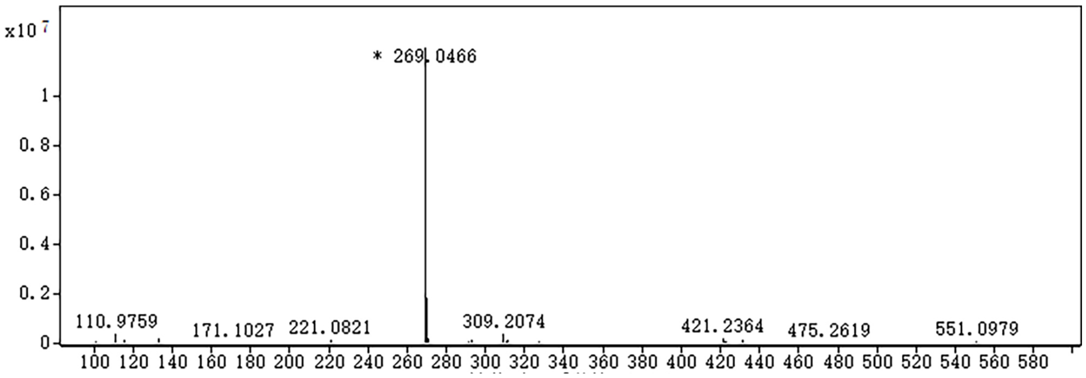

Figure S2 HRESIMS spectrum of emodin
